# STMN1 Promotes Progesterone Production Via StAR Up-regulation in Mouse Granulosa Cells

**DOI:** 10.1038/srep26691

**Published:** 2016-06-08

**Authors:** Yun-De Dou, Han Zhao, Tao Huang, Shi-Gang Zhao, Xiao-Man Liu, Xiao-Chen Yu, Zeng-Xiang Ma, Yu-Chao Zhang, Tao Liu, Xuan Gao, Lei Li, Gang Lu, Wai-Yee Chan, Fei Gao, Hong-Bin Liu, Zi-Jiang Chen

**Affiliations:** 1 Center for Reproductive Medicine, Shandong Provincial Hospital Affiliated to Shandong University, Jinan, China; 2 National Research Center for Assisted Reproductive Technology and Reproductive Genetics, China; 3 The Key laboratory for Reproductive Endocrinology of Ministry of Education, China; 4 Shandong Provincial Key Laboratory of Reproductive Medicine, Jinan, China; 5 The Chinese University of Hong Kong-Shandong University Joint Laboratory on Reproductive Genetics, School of Biomedical Sciences, The Chinese University of Hong Kong, Hong Kong SAR, China; 6 State Key Laboratory of Reproductive Biology, Institute of Zoology, Chinese Academy of Sciences, Beijing, China; 7 Center for Reproductive Medicine, Ren Ji Hospital, School of Medicine, Shanghai Jiao Tong University, Shanghai, China; 8 Shanghai Key Laboratory for Assisted Reproduction and Reproductive Genetics, Shanghai, China

## Abstract

Stathmin 1 (STMN1) is a biomarker in several types of neoplasms. It plays an important role in cell cycle progression, mitosis, signal transduction and cell migration. In ovaries, STMN1 is predominantly expressed in granulosa cells (GCs). However, little is known about the role of STMN1 in ovary. In this study, we demonstrated that STMN1 is overexpressed in GCs in patients with polycystic ovary syndrome (PCOS). In mouse primary GCs, the overexpression of STMN1 stimulated progesterone production, whereas knockdown of STMN1 decreased progesterone production. We also found that STMN1 positively regulates the expression of *Star* (steroidogenic acute regulatory protein) and *Cyp11a1* (cytochrome P450 family 11 subfamily A member 1). Promoter and ChIP assays indicated that STMN1 increased the transcriptional activity of *Star* and *Cyp11a1* by binding to their promoter regions. The data suggest that STMN1 mediates the progesterone production by modulating the promoter activity of *Star* and *Cyp11a1*. Together, our findings provide novel insights into the molecular mechanisms of STMN1 in ovary GC steroidogenesis. A better understanding of this potential interaction between STMN1 and *Star* in progesterone biosynthesis in GCs will facilitate the discovery of new therapeutic targets in PCOS.

Polycystic ovary syndrome (PCOS) is one of the most common but heterogeneous endocrine metabolic disorders in 6% to 8% of Asian women of reproductive age and causes abnormal ovulation and infertility^1^,^2^. The characteristic clinical features of PCOS include oligomenorrhea or amenorrhea, hyperandrogenism, and polycystic ovarian morphology^3^. PCOS has been attributed to hypothalamic–pituitary disorders, aberrant gonadotropin secretion, dysfunction of theca and/or granulosa cells (GCs) and different types of metabolic disorders, including ovarian androgen overproduction, hyperinsulinemia, and insulin resistance^4^,^5^. Yet, the exact pathophysiology needs to be examined^6^.

STMN1 is a highly conserved gene that codes for cytoplasmic phosphoproteins. As a small regulatory protein, it integrates diverse intracellular signaling pathways involved in the control of cell proliferation, differentiation, and other activities^7^. STMN1 promotes depolymerization of microtubules and/or prevents polymerization of tubulin heterodimers, and thereby plays a critically important role in the regulation of the cell cycle^8^. STMN1 also plays a potential role in the regulation of hormone secretion in rodent pituitary and insulinoma cell lines^8^. Furthermore, STMN1 mediates nerve growth factor (NGF)-induced GCs apoptosis through tumor necrosis factor α (TNF-α). NGF promotes steroidogenesis by enhancing the expression of enzymes involved in progesterone, testosterone, and estradiol (E2) synthesis^9^, indicating that STMN1 might as well be involved in the regulation of gonadal hormones and associated diseases in the reproductive system. At the present time, however, little is known about the potential role of STMN1 in ovarian function, such as follicular development, oocyte maturation, and ovarian steroidogenesis Table 1.

We found that STMN1 was highly expressed in GCs of PCOS patients and it could promote the synthesis of progesterone in mouse primary GCs *in vitro*. In most mammals, progesterone is a key steroid hormone in the maintenance of normal pregnancy and regulation of estrus cycle. Progesterone is synthesized by the corpus luteum (CL), which is a transient endocrine gland originating in ovulating follicles^10^. Progesterone is synthesized from cholesterol, which is present in the blood as low density lipoprotein (LDL) and transferred to cells by receptor-mediated endocytosis^11^. Steroidogenic acute regulatory protein (StAR) controls the process of cholesterol transport from the outer to inner mitochondrial membrane, where cytochrome P450 side chain cleavage enzyme (P450scc) catalyzes the first step in progesterone synthesis^12^. The resulting pregnenolone is then converted to progesterone by 3β-hydroxysteroid dehydrogenase (3βHSD) in the smooth endoplasmic reticulum^13^. The role of STMN1 in mouse GCs steroidogenesis remains to be examined, and the related molecular mediators involved in this process have yet to be defined. In this study, we investigated the role of STMN1 in progesterone biosynthesis and the underlying mechanisms in mouse primary GCs.

## Results

### *STMN1* mRNA is highly expressed in granulosa cells of PCOS patients

STMN1 was previously reported to be involved in TNF/STMN1-mediated GC death pathway in response to excessive ovarian NGF production^9^. Since GCs plays an important role in normal follicular development, abnormal GCs functions may lead to follicular development disorder. It is known that PCOS ovaries contain twice the number of growing follicles compared to normal ovaries at all stages of development^14^ and GC proliferation is altered in PCOS^15^. To investigate whether STMN1 participates in the pathophysiology of PCOS, we determined STMN1 expression in human ovarian GCs.

**Figure 1:**
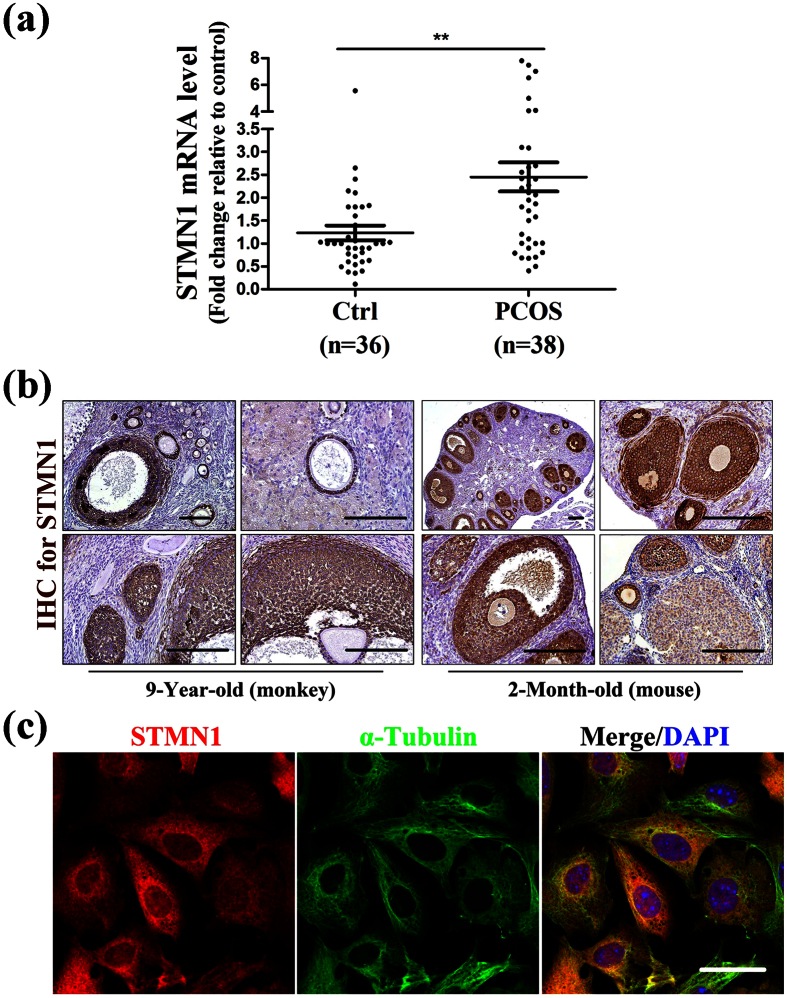
STMN1 mRNA levels in GCs in women with or without PCOS and its localization within ovaries. (**a**) A total of 38 unrelated Han Chinese women with PCOS and 36 normal women under IVF-ET therapy were recruited. The expression level of housekeeping gene, *Actb*, was used to normalize gene expression. Quantitative real-time RT-PCR demonstrated that STMN1 expression level in GCs of PCOS was nearly two folds higher than normal women. **P < 0.01. (**b**) Immunohistochemistry of rhesus monkey and mouse ovary using anti-STMN1 antibody (Abcam) showed that STMN1 was expressed exclusively in GCs of follicles at various stages, including primary, secondary, and mature follicles, and expressed little in oocytes, which stained positive (yellow). Scale bar = 100 μm. (**c**) Immunofluorescence assay of primary GCs in mouse using anti-STMN1 antibody (Abcam). STMN1 expressed in the cytoplasm and nucleus. Scale bar = 20 μm.

STMN1 mRNA was over-expressed in the GCs of PCOS patients. Specifically, STMN1 mRNA level in women with PCOS was nearly two-fold higher compared to the control subjects (Fig. 1a), indicating that STMN1 may be involved in the pathophysiology of PCOS, especially in GCs’ function. The demographics and basic characteristics of the patients are shown in Table 1.

### STMN1 localizes to granulosa cells of follicles

Although STMN1 was previously reported to be mostly expressed in GCs of mouse antral follicles^9^, it remains unclear where STMN1 localizes within the whole ovary. We examined STMN1 expression in ovary by immunohistochemistry in monkey and mouse ovaries, respectively (Fig. 1b). Immunohistochemistry revealed that STMN1 was exclusively expressed in GCs of follicles at various stages, including primary, secondary, and mature follicles, and expressed minimally in oocytes.

STMN1 has been reported to be a widespread small regulatory protein, which mainly localizes in the cytoplasm^7^. It also distributes in cell nucleus^16^. To investigate the intracellular expression of STMN1, immunofluorescence was performed using mouse primary GCs. The results showed that STMN1 (stained red) was expressed in the cytoplasm, as well as in the nucleus (Fig. 1c).

### STMN1 regulates progesterone synthesis in GCs

To investigate the possible effect of STMN1 on GCs function, the *in vitro* culture model of mouse primary GCs was employed. Adeno virus and siRNA pool targeting STMN1 were used to overexpress or knockdown STMN1 expression. The efficacy was validated on mRNA level. At 48 h and 72 h of post-treatment, the GCs were harvested for western blot or real-time RT-PCR and the culture media were collected for hormone analysis.

**Figure 2:**
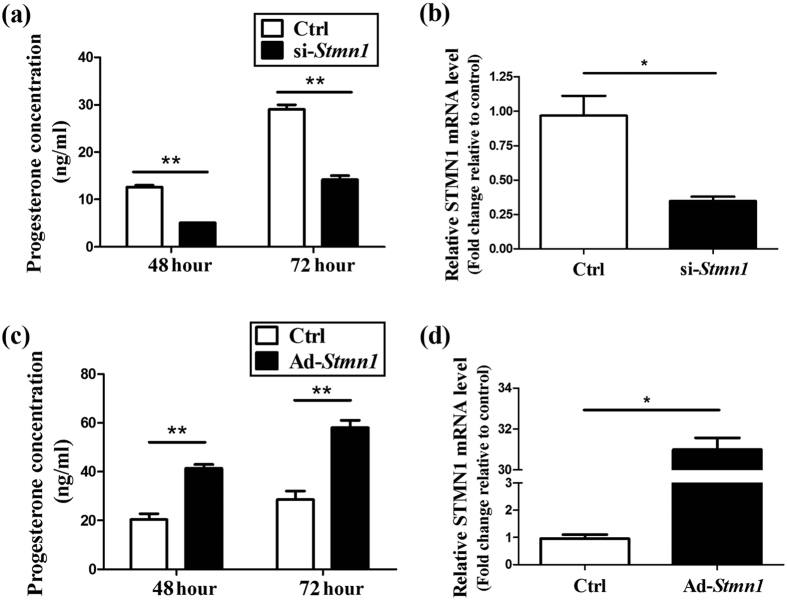
STMN1 may up-regulate the progesterone production of ovarian GCs. (**a**) Knockdown of STMN1 decreased the progesterone levels in mouse primary GCs culture medium. Note the decrease of progesterone production after Ad-*Stmn1* treatment to 50% of the control level. (**b**) STMN1 was down-regulated by 30% by 25 nM si-*Stmn1*. (**c**) Up-regulation of STMN1 increased the concentration of progesterone in GCs culture medium. Note the increase of progesterone production after Ad-*Stmn1* treatment to two-fold of control level. (**d**) STMN1 was up-regulated by 32 fold by Ad-*Stmn1*. Data shown represent the mean ± SEM of three independent experiments performed in triplicate. *P < 0.05, **P < 0.01.

Knockdown of STMN1 with siRNA pool resulted in decreased progesterone production in the media (Fig. 2a). The control progesterone levels at 48 h and 72 h were 11.15 ± 1.15 ng/mL and 29.00 ± 1.41 ng/mL respectively, whereas those of down-regulated GCs were 6.58 ± 0.43 ng/mL and 14.23 ± 0.78 ng/mL respectively. In contrast, overexpression of STMN1 led to increased progesterone production in the culture media (Fig. 2c). The progesterone concentration of controls at 48 h and 72 h were 20.37 ± 3.35 ng/mL and 28.50 ± 4.95 ng/mL respectively, whereas those of up-regulated GCs were 41.35 ± 2.33 ng/mL and 58.00 ± 4.24 ng/mL respectively. These results suggest that STMN1 may up-regulate the progesterone production of ovarian GCs.

### STMN1 produces no significant effect on GCs’ proliferation *in vitro*

**Figure 3:**
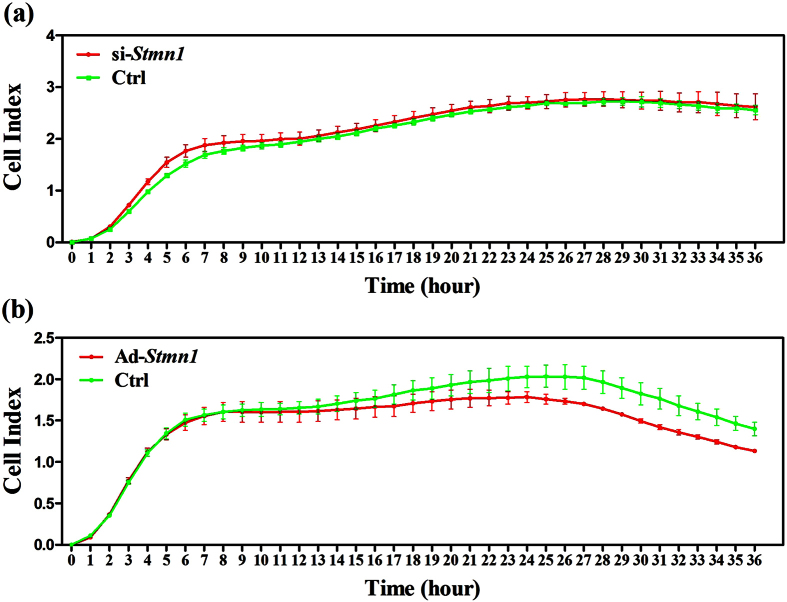
Effect of STMN1 on mouse primary GCs’ proliferation monitored by Roche xCelligence in real time. Mouse primary GCs were treated with specific siRNA and adenovirus respectively. Cell indices were plotted against cultured time as growth curves. (**a**) STMN1 knockdown did not significantly alter GCs proliferation. (**b**) Up-regulation of STMN1 slightly inhibited the proliferation of mouse GCs.

Given the fact that STMN1 is involved in the control of cell proliferation^7^, STMN1 could affect the proliferation of GCs. In its phosphorylated state, STMN1 mediates a GC death signal pathway initiated by the NGF, for example, excessive NGF increases its abundance as well as its forms of phosphorylation at serine (Ser) 16, 25, and 38^9^. To investigate the possible effect of STMN1 on GCs’ proliferation, analysis of cell proliferation was performed using Xcelligence real-time cellular analysis system. The results revealed that STMN1 knockdown produced no obvious effect on GCs proliferation (Fig. 3a), whereas up-regulation of STMN1 slightly inhibited GCs proliferation (Fig. 3b).

### STMN1 promotes progesterone accumulation by inducing *Star* and *Cyp11a1* expression

The effect of STMN1 on GCs’ proliferation does not explain the altered progesterone levels. Therefore, we investigated whether the elevated progesterone resulted from the altered expression of genes encoding steroidogenic enzymes involved in the synthesis of progesterone.

**Figure 4:**
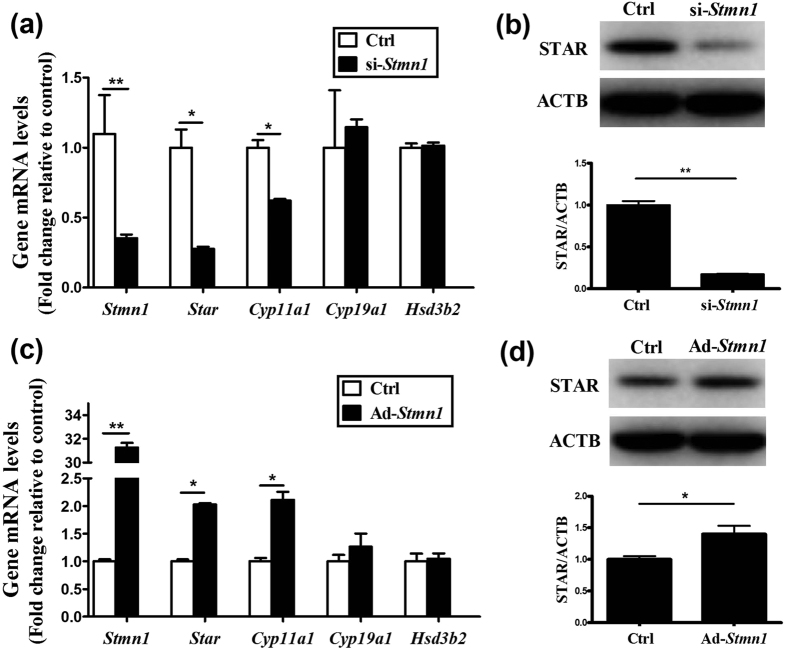
Effect of STMN1 on ovarian GC steroidogenesis-related genes. (**a**,**c**) Quantitative real-time RT-PCR analysis of ovarian GC steroidogenesis-related gene expression. GCs were transfected with Ad-control (siRNA-control) or Ad-*Stmn1* (siRNA-*Stmn1*) and total mRNA was extracted and subjected to real-time PCR and data obtained from Ad-control (siRNA-control) transfected cells were set at 1.0. (**b,d**) Western blot analysis of StAR expression. After treatment for 48 hours, the GCs lysates were analyzed by immunoblotting with anti-StAR (ProteinTech Group) at 1:1000 dilution and anti-ACTB (ProteinTech Group) at 1:1000 dilution. ACTB was used as a loading control. *P < 0.05, **P < 0.01.

Transfection of GCs with siRNA-*Stmn1* down-regulated the mRNA levels of *Star* and *Cyp11a1* (Fig. 4a). Furthermore, up-regulation of STMN1 using adenovirus induced the expression of *Star* and *Cyp11a1* mRNA (Fig. 4c). Western blot assay confirmed the changes of StAR protein levels (Fig. 4b,d), but no obvious change of CYP11A1 was detected.

### STMN1 binds *Star* promoter and triggers gene transcription

**Figure 5:**
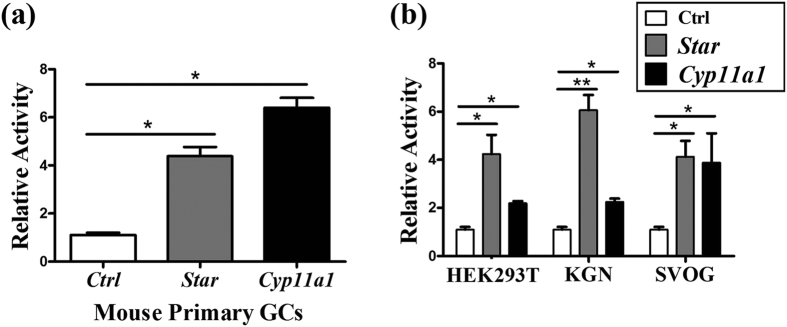
STMN1 activates *Star* and *Cyp11a1* transcription. (**a**) In mouse primary GCs, STMN1 enhanced the transcriptional activities of *Star* and *Cyp11a1* promoters four- and seven-fold. (**b**) To diminish the endogenous STMN1 effect, HEK293T, KGN and SVOG cells were also used, which confirmed the analogous results in mouse primary GCs. *P < 0.05, **P < 0.01.

To detect the possible effect of STMN1 on promoter activities of *Star* and *Cyp11a1*, dual-luciferase reporter gene assay was performed. STMN1 overexpression resulted in approximately 4-fold induction of *Star* promoter activity and 7-fold induction of *Cyp11a1* promoter activity compared with negative control (Fig. 5a). To diminish the effect of endogenous STMN1, HEK293T, KGN and SVOG cells were used, which confirmed the analogous results in mouse primary GCs (Fig. 5b).

**Figure 6:**
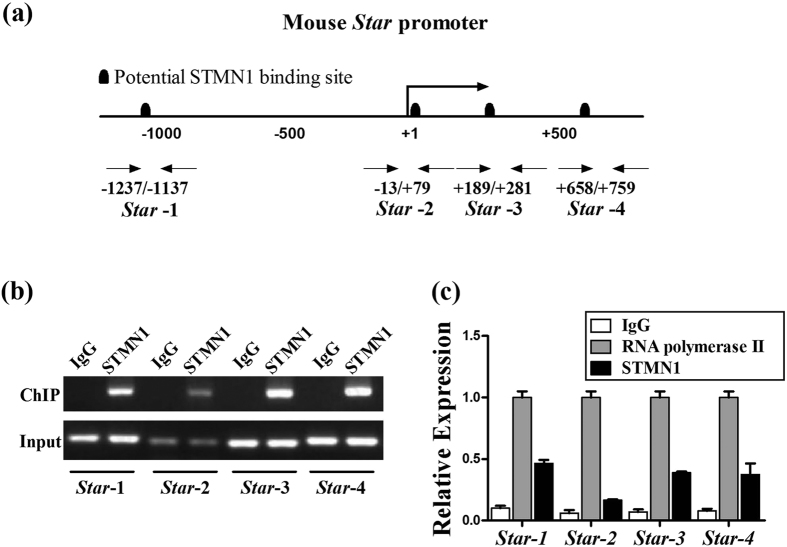
STMN1 binding to the upstream region of the mouse *Star* gene. (**a**) Representation of potential STMN1-binding motifs on the mouse *Star* promoter and five primer pairs designed for ChIP quantitative PCR; (**b**) STMN1 bound the *Star* promoter. Normalized inputs of GCs chromatin DNA were pulled down by STMN1 or negative IgG antibodies. The DNA template was amplified by PCR using primer pairs 1-5 against the possible binding sites. (**c**) Ratios of the “ChIP band” to the “input band”. IgG controls were normalized to 1.0. ChIP experiments and PCR reactions were repeated twice, and quantified as the mean ± SEM.

Since STMN1 also distributes in cell nucleus, to examine the possible interaction of STMN1 and promoter, ChIP assay was performed using mouse primary GCs. We screened the promoters of *Star* with Genomatix MatInspector and detected several potential STMN1-binding sites (Fig. 6a). The data suggested that *Star* may act as a direct STMN1 target gene. The subsequent ChIP assay revealed that *Star*-1 (−1,196/−1,177), *Star*-3 (+247/+269) and *Star*-4 (+71/+741) were strongly amplified compared to negative controls, indicating specific STMN1 binding to the *Star* promoter regions (Fig. 6b,c). The predicted binding sites and results of ChIP assay about *Cyp11a1* can be found as Supplementary Figure S3.

## Discussion

In this study, we demonstrated the differences in STMN1 mRNA levels in GCs in normal and PCOS women. STMN1 promoted progesterone production by inducing StAR in mouse primary GCs. Specifically, STMN1 mRNA expression level in GCs of women with PCOS was nearly two folds higher than normal women. Furthermore, STMN1 enhanced the rate-limiting regulatory protein StAR by affecting its promoter activity via direct binding, and thus affected the progesterone accumulation in mouse primary GCs *in vitro*.

STMN1 continues to attract profound research interest because of its probable role in transcription or posttranscriptional modification. Previous studies indicated an elevated STMN1 expression across a broad range of human malignancies^17^. Interestingly, STMN1 is also involved in GC apoptosis in response to excessive ovarian NGF production^9^. In this study, we further investigated the expression of STMN1 in GCs in various stages of follicles and demonstrated highly expressed STMN1 mRNA level in PCOS patients’ GCs. The data suggest that STMN1 may be involved in the pathophysiology of PCOS.

PCOS patients exhibit no detectable or very low levels of progesterone due to irregular menses and anovulatory cycles. However, serum hormone levels of the classic endocrine theory may not reflect the real conditions of follicles, which are a relatively independent paracrine and autocrine microenvironment^18^. The follicular microenvironment may have more direct effects on follicular development, ovulation and luteinization^19^. Hence, analyzing the progesterone levels in follicular fluid may be more informative than the studies of serum in the context of ovarian reproductive disorders, infertility and assisted reproductive technology. Some previous studies demonstrated significantly reduced follicular fluid progesterone levels in PCOS compared to normal subjects^20^,^21^. In contrast, another study with a larger sample pool showed that patients with PCOS had similar follicular fluid progesterone concentrations compared with volume-matched control follicles^22^. It is of particular interesting, however, there is evidence that the progesterone concentration was higher in follicles with meiotically incompetent oocytes from women with PCOS compared to those in normal ovaries^23^. Furthermore, the progesterone concentration was correlated with oocyte quality in this study^23^. The underlying mechanisms are unknown and more studies are required to investigate specific differences in follicular fluid progesterone levels in PCOS and non-PCOS women. It is possible that PCOS patients may have higher follicular fluid progesterone levels, along with the expression of STMN1. High progesterone and STMN1 expression were found in ovarian cancer types, such as ovarian granular cell carcinoma^24^. This suggests that elevated STMN1 expression may be a cause for high progesterone apart from GCs proliferation.

Following ovulation, GCs become a part of corpus luteum and generate large quantities of progesterone to establish and maintain early pregnancy. However, elevated progesterone levels prior to ovulation can lead to premature luteinization, which adversely affects oocyte quality and endometrial receptivity^25^,^26^. Although pituitary gonadotropins are key regulators of luteal steroidogenesis^27^, other factors such as IGF and TGF-β also play important roles in paracrine or autocrine^28^,^29^. In this study, we demonstrated that the combination of STMN1 and promoter regions of *Star* and *Cyp11a1* induced progesterone levels in PND 12 mouse ovarian GCs. However, no analogous result was found when using the GCs of PND 24-26 mouse treated with Pregnant Mare Serum Gonadotropin (PMSG) for 24 h. This suggests that STMN1 may play its role in the procedure of GCs luteinization.

Steroid hormone biosynthesis begins with the enzymatic conversion of cholesterol to pregnonolone catalyzed by the P450scc^30^. One of the critical rate-limiting steps in the process is the delivery of the main substrate to the inner mitochondrial membrane^31^. And StAR is a key factor that facilitates the translocation of cholesterol from cellular stores across the aqueous intermembrane space of the mitochondria to the inner membrane^32^. StAR is regulated by hormones and other factors involved in protein synthesis. The regulators include developmental factors, such as LH, FSH, GH, and ACTH, and transcription factors, such as SF-1, C/EBPs, Sp1, and DAX-1^33^. In the human ovary, the expression of StAR is regulated throughout the luteal phase and it plays a vital role in controlling progesterone production during the development and demise of the corpus luteum^34^. We demonstrated that STMN1 transactivates the StAR expression by binding to the promoter region of *Star* and, which in turn, induces the progesterone production in mouse ovarian GCs. Interestingly, StAR expression was also elevated in the ovary of rat^35^ and sheep^36^ PCOS models induced by prenatal hyperandrogenism or letrozole^37^.

STMN1 controls cell cycle^8^ and additional studies that investigate the relationship between GCs cell cycle and progesterone synthesis are underway. The STMN1 phosphorylation and mutational analysis in PCOS will be required to future demonstrate the role of STMN1.

## Methods

### Subjects

We recruited patients from the Reproductive Hospital Affiliated to Shandong University, between January and June of 2014. Anthropometric variables, such as age, height, body weight, menstrual cycle, and select endocrine and biochemical parameters were recorded. A total of 38 unrelated Han Chinese women with PCOS were selected in strict accordance with the Rotterdam criteria, which requires the presence of at least two of the following criteria for PCOS diagnosis: oligo- and/or anovulation; clinical and/or biochemical signs of hyperandrogenism; and polycystic ovaries with exclusion of other causes of hyperandrogenism, such as hyperprolactinemia, androgen-secreting tumors, Cushing’s syndrome, and non-classical congenital adrenal hyperplasia^38^. And 36 additional unrelated Han Chinese women who were free of hormone therapy for at least three months^39^ and with regular menstrual cycles (26–35 days), normal ovarian morphology, and normal hormone levels were included as controls. The controls were treated with IVF-ET therapy because of infertility caused by reasons other than PCOS. Written informed consent was obtained from each subject.

### Isolation and culture of cells

Mouse primary ovarian GCs were isolated from preantral follicles (120–150 μm in diameter) of postnatal day 12 mouse obtained from the Laboratory Animal Center, Shandong University as previously described^40^. Proliferation of GCs in follicles of this size range was the most active^41^. This granulosa cell isolation integrates mechanical method and digesting method and is relatively blameless which has been usually used in the granulosa cells’ research^42^,^43^. Briefly, the mice were killed by cervical dislocation, the ovaries excised, and the follicles were isolated with insulin syringe fine needles. The follicles were then shredded and treated with type I collagenase (Worthington Biochemical Corporation) and recombinant type I deoxyribonuclease (Worthington Biochemical Corporation). The digested GCs were washed and collected by brief centrifugation, and then cultured in DMEM/F12 (HyClone corporation, Utah, USA) supplemented with 10% fetal bovine serum (FBS) (HyClone corporation) and 1% antibiotics (100 U/mL penicillin and 100 μg/mL streptomycin; HyClone corporation). The cells were used for experiments at the second passage.

The HEK293T cell line was grown in DMEM High Glucose (HyClone corporation) supplemented with 10% FBS and 1% antibiotics. The SVOG (gifted from Prof. Peter C.K. Leung of University of British Columbia), an immortalized human granulosa cell line obtained from women undergoing IVF therapy via being transfected with the SV40 large T antigen^44^, and KGN (obtained from RIKEN BioResource Center (Ibaraki, Japan)), a steroidogenic human granulosa-like tumor cell line^45^, were grown in DMEM/F12 supplemented with 10% FBS and 1% antibiotics. All the cells were cultured in a humidified atmosphere containing 5% CO_2_ and 95% air at 37 °C.

### Immunohistochemistry and immunofluorescence

Immunohistochemical analysis was performed on ovarian sections from 9-year-old rhesus monkey and 2-month-old mouse in order to determine the localization of STMN1. Briefly, the ovaries were fixed with 4% paraformaldehyde for 24 h, and then washed with PBS and stored in 70% ethanol. The samples were embedded in paraffin, and 5-μm sections were prepared. After deparaffinization and rehydration through a graded ethanol series, the slides were incubated with 5% BSA for 30 min at room temperature and incubated with anti-STMN1 (Abcam, Cambridge, MA) antibody at 1:100 dilution overnight at 4 °C. After washing with PBS, the sections were incubated with biotinylated secondary antibody (Abcam) for 1 h. The slides were then incubated with avidin-biotin-peroxidase complex for 1 h at room temperature. Reaction color was developed with 3,3-diaminobenzidine for 2 min and counterstained with hematoxylin for 2 min.

In immunofluorescence assay, the cultured GCs were fixed with 4% paraformaldehyde and blocked with 5% BSA. Then cells were incubated with anti-STMN1 antibody (Abcam, Cambridge, MA) at 1:100 dilution overnight at 4 °C. Then, cells were incubated with fluorescence-labeled secondary antibody for 1 h.

### Adenoviral vector construction

The recombinant adenovirus was obtained from Genechem (Shanghai, China) using the AdMax system (Microbix, Canada). Briefly, the cDNA sequence of *Stmn1* (NM_019641) was cloned by reverse transcription polymerase chain reaction (RT-PCR), and then subcloned into GV135 vector (Genechem, Shanghai, China). The GV135-*Stmn1* was recombined with pBHG lox ΔE1,3 Cre plasmid (Microbix) in HEK293T cells. Concurrently, the adenovirus of AdGV135 was generated using “empty” GV135 vector as the control.

### siRNA and transfection

ON-TARGET *plus* SMART *pool* siRNA targeting *Stmn1* and CONTROL NON-TARGETING *pool* siRNA (Dharmacon, GE Healthcare Life Technologies) were transfected at 50 nM. Primary GCs were seeded in normal growth medium to attain 60–70% confluence, and then incubated in antibiotic-free DMEM/F12 medium containing 10% FBS for 3–4 h before transfection. Cells were transfected with siRNA using X-tremeGENE siRNA Transfection Reagent (Roche, Penzberg, Germany) for 24 h or 48 h.

### Real-time RT-PCR

Total RNA was extracted from cultured GCs using TRIzol reagent (Takara Bio Inc., Dalian, China) and was reversely transcribed into cDNA using PrimeScript RT reagent Kit With gDNA Eraser (Takara Bio Inc.). The real-time polymerase chain reactions were performed using SYBR Premix Ex Taq (Takara Bio Inc.) according to the manufacturer’s instructions. The primers can be found as Supplementary Table S1. Real-time PCRs were carried out using Roche LightCycle 480 (Roche, Penzberg, Germany). The expression of the housekeeping gene, *Actb* (also known as *β-Actin*), was used to normalize gene expression. The relative level of gene transcripts was calculated on the basis of CP value using the comparative cycle threshold method.

### Western blot analysis

After treatment, GCs were harvested and lysed in RIPA buffer containing 1 mM phenylmethylsulfonyl fluoride (PMSF). Equal amounts of protein were electrophoresed on 10% sodium dodecyl sulfate polyacrylamide gel (SDS-PAGE), and the bands were transferred to polyvinylidene fluoride membrane (Millipore, USA). The membrane was blocked and then incubated with the relevant primary antibodies. After washing, the membranes were incubated with peroxidase-conjugated secondary antibodies (Zhongshan, Beijing, China) for 1 h. Immunoreactive bands were detected and analyzed with BIO-RAD ChemiDoc MP Imaging System and Image Lab Software. Relative protein levels in each sample were normalized to ACTB to standardize the loading variations.

### Proliferation assay

Mouse primary GCs were cultured in phenol red-free DMEM (GIBCO, Invitrogen, Carlsbad, CA, USA) supplemented with 5% FBS. After treatment with siRNA or adenovirus, cells were passaged to the special culture plate at a density of 15,000 cells per well for real-time cell proliferation assay using the Xcelligence real-time cellular analysis SP instrument (Roche Applied Science, Penzberg, Germany)^46^. The growth curves (cell indices vs. culture time) were automatically recorded in the xCELLigence System (Roche Applied Sciences) in real time.

### Luciferase reporter assay

Luciferase assays were performed using the Dual-Luciferase Reporter Assay System (Genecopoeia, Guangzhou, China). The custom GLuc-ON reporter construct (GeneCopoeia) containing the *Star* promoter (fragment spanning nucleotides −1,389 to +50) or *Cyp11a1* promoter (fragment spanning nucleotides −1,377 to +3) sequence cloned in front of the *Gaussia* luciferase gene was co-transfected to the GCs with the *Stmn1* expression vector (*Stmn1*-pCDH) or control vector (pCDH) using X-tremeGENE HP DNA transfection reagent (Roche, Penzberg, Germany). Luciferase activities of cultured supernatant were measured 48 h and 72 h after transfection using a Secrete-Pair™ Dual Luminescence Assay Kit (Genecopoeia) according to the manufacturer’s instructions. The *Gaussia* luciferase (GLuc) activity was normalized to Secreted Alkaline Phosphatase luciferase (SEAP) activity for transfection efficiency. The activity ratio of the empty vectors was arbitrarily set at 1.0. Each value represents the mean for at least three independent experiments.

### Hormone analysis

After the specified treatments, the culture medium was assayed immediately or stored at −80 °C until assayed. The progesterone concentration in the conditioned medium was diluted to one twentieth with saline and then measured using the Access Immunoassay System (Beckman Coulter, Inc., Brea, CA), according to the manufacturer’s instructions. The inter- and intra-assay coefficients of variation for this assay were less than 10%. Each sample was measured in triplicate.

### ChIP assay

ChIP assays were performed as described^47^ using Pierce Agarose ChIP Kit (ThermoFisher, USA) according to the manufacturer’s instructions. The cell pellets were suspended in lysis buffer and vortexed to shear DNA. After vortexing, the lysate was centrifuged, and the supernatant was diluted 10-fold with ChIP dilution buffer. Rabbit anti-STMN1 (Abcam) and rabbit anti-RNA polymerase II (Bioss, China) or normal rabbit IgG (negative control) were added to the supernatant and incubated overnight at 4 °C with rotation. The immunocomplex was precipitated with protein A/G-agarose, washed, and eluted with elution buffer. Reversal of cross-linking was performed by heating at 65 °C overnight in the presence of NaCl. The promoter regions of the mouse *Star* and *Cyp11a1* promoter, which contain putative STMN1-binding sites, were amplified using specific primers (See Supplemental Table S2).

### Statistical analysis

Data were expressed as the Mean ± SEM from at least three independent experiments. One-way ANOVA followed by Tukey’s multiple comparison tests were used for statistical comparison. Values were considered significant at *P* < 0.05.

### Ethics statement

This study received ethical approval of the Institutional Review Board of Reproductive Medicine of Shandong University (Jinan, China). All the methods described here were carried out in accordance with the guidelines and regulations approved by the Institutional Review Board of Reproductive Medicine of Shandong University.

## Additional Information

**How to cite this article**: Dou, Y.-D. *et al.* STMN1 Promotes Progesterone Production Via StAR Up-regulation in Mouse Granulosa Cells. *Sci. Rep.* **6**, 26691; doi: 10.1038/srep26691 (2016).

## Supplementary Material

Supplementary Information

## Figures and Tables

**Table 1:** Clinical Profile of Subjects with PCOS and Controls.

	PCOS	Controls
No.	38	36
Age (years)	28.08 ± 2.32	28.89 ± 2.67
BMI (kg/m^2^)	23.95 ± 3.30	22.61 ± 3.26^a^
LH (IU/L)	13.06 ± 5.80	4.85 ± 1.85^a^
Testosterone (ng/dL)	45.92 ± 15.88	20.17 ± 6.99^a^
Progesterone (ng/mL)	1.10 ± 2.37	0.54 ± 0.21
FSH (IU/L)	6.00 ± 1.23	6.78 ± 1.27^a^
PRL (ng/mL)	15.93 ± 8.14	14.28 ± 5.40
E2 (pg/mL)	44.20 ± 17.03	32.14 ± 10.60^a^
TSH (μIU/mL)	2.66 ± 2.04	3.61 ± 5.75

BMI: body mass index. FSH: follicle stimulating hormone. LH: luteinizing hormone. T: testosterone. Data shown represent the mean ± SD. The BMI, LH, Testosterone, FSH and E2 levels of the PCOS women are significantly different than those of the normal women. The hormone values are measured in base levels. ^a^*P* < 0.05.
