# Supplementary material for: STMN1 Promotes Progesterone Production Via StAR Up-regulation in Mouse Granulosa Cells
Source: Sci Rep. 2016 Jun 8;6:26691. doi: 10.1038/srep26691 (PMC4897624; doi:10.1038/srep26691)

**Title：**STMN1 Promotes Progesterone Production Via StAR Up-regulation in Mouse Granulosa Cells

**Authors’ names and institutions**:

**Yun-De Dou^1,*^, Han Zhao^1,*^, Tao Huang^1^, Shi-Gang Zhao^1^, Xiao-Man Liu^1^, Xiao-Chen Yu^1^, Zeng-Xiang Ma^1^, Yu-Chao Zhang^1^, Tao Liu^1^, Xuan Gao^1^, Lei Li^1^, Gang Lu^3^, Wai-Yee Chan^3^, Fei Gao^2^, Hong-Bin Liu^a,1,3^, Zi-Jiang Chen^b,1,4^**

^1^Center for Reproductive Medicine, Shandong Provincial Hospital Affiliated to Shandong University, Jinan, China; National Research Center for Assisted Reproductive Technology and Reproductive Genetics, China; The Key laboratory for Reproductive Endocrinology of Ministry of Education, China; Shandong Provincial Key Laboratory of Reproductive Medicine, Jinan, China.

^2^State Key Laboratory of Reproductive Biology, Institute of Zoology, Chinese Academy of Sciences, Beijing, China.

^3^The Chinese University of Hong Kong-Shandong University Joint Laboratory on Reproductive Genetics, School of Biomedical Sciences, The Chinese University of Hong Kong, Hong Kong SAR, China.

^4^ Center for Reproductive Medicine, Ren Ji Hospital, School of Medicine, Shanghai Jiao Tong University, Shanghai, China; Shanghai Key Laboratory for Assisted Reproduction and Reproductive Genetics, Shanghai, China.

**Correspondence to: ^a^**Hong-Bin Liu ([humangenetics2008@hotmail.com](mailto:humangenetics2008@hotmail.com)) or ^b^Zi-Jiang Chen ([chenzijiang@hotmail.com](mailto:chenzijiang@hotmail.com) )

*These authors contributed equally to this work.

**Supplemental table S1: RT-PCR Oligonucleotide Primers**

| Gene name | GenBank |  | Primer sequences (5’ -3’) | Size (bp) |
| --- | --- | --- | --- | --- |
| *Stmn1* | NM_019641 | Forward | CCAGGCTTTTGAGCTGATTC | 133 |
|  |  | Reverse | GCGTCTTTCTTCTGCAGCTT |  |
| *Star* | NM_011485 | Forward | TTGGGCATACTCAACAACCA | 103 |
|  |  | Reverse | GAAACACCTTGCCCACATCT |  |
| *Cyp11a1* | NM_019779 | Forward | GGCACTTTTGGAGTCAGTTTACAT | 187 |
|  |  | Reverse | GTTTAGGACGATCTGGTCTTTCTT |  |
| *Cyp19a1* | NM_007810 | Forward | ATTCTTGTGGATGGGGATTG | 159 |
|  |  | Reverse | CCGAATCGGGAGATGTAGTG |  |
| *Hsd3b2* | NM_153193 | Forward | TCCAGCTCAGTTGATGTTGC | 129 |
|  |  | Reverse | TGCCTTCTCAGCCATCTTTT |  |
| *Actb* | NM_007393 | Forward | CATCCGTAAAGACCTCTATGCCAAC | 171 |
|  |  | Reverse | ATGGAGCCACCGATCCACA |  |
| *STMN1* | NM_203401 | Forward | ATTCTCAGCCCTCGGTCAAA | 172 |
|  |  | Reverse | TCTCGTGCTCTCGTTTCTCA |  |
| *ACTB* | NM_001101 | Forward | CATCGAGCACGGCATCGTCA | 211 |
|  |  | Reverse | TAGCACAGCCTGGATAGCAAC |  |

**Supplemental table S2: Oligonucleotide Primers Used in ChIP Assay**

| Gene |  | Primer sequences (5’ -3’) | Size (bp) |
| --- | --- | --- | --- |
| *Star*-1 | Forward | GCAGGGTCTGGTGCAGAGC | 100 |
|  | Reverse | CTTCTTTCCAAGTACCTTTGAGGCTAC |  |
| *Star*-2 | Forward | CTCAGCACTCAGCATGTTCCTC | 92 |
|  | Reverse | CCAACCCAGACTCACCTTTCAT |  |
| *Star*-3 | Forward | CTGGGGGAGCCTGGGT | 92 |
|  | Reverse | TTGGATGAGAGGTGGCACC |  |
| *Star*-4 | Forward | GTCTGACCAGCCCACCCC | 101 |
|  | Reverse | GCCACATCTCTAACTCTCAAGTTCAGTG |  |
| *Cyp11a1*-1 | Forward | CGTCAATCCCAGCATTCAGG | 77 |
|  | Reverse | CAGCTAACCTGGAACTCTCTATGTAGATC |  |
| *Cyp11a1*-2 | Forward | CACACGGCAGCTCAACTATCTG | 102 |
|  | Reverse | GTTGTTTTTTTATCTATTTAATGTGCGTTG |  |
| *Cyp11a1*-3 | Forward | CTCTCATCTTAGAGGATATTCGTCATCAC | 100 |
|  | Reverse | CTCTGCAATAGTTGGGTTTGCTTC |  |
| *Cyp11a1*-4 | Forward | CGACAATGGTTGGCTAAACCTG | 93 |
|  | Reverse | CCATACTTTTGGAAACTCTGCATCTG |  |
| *Cyp11a1*-5 | Forward | GCCTTTTTCTTTCATTTCTTCCAGG | 169 |
|  | Reverse | CTTGGAGTCAGAAATGAGGGAAGACTAT |  |

**Supplemental figure S3: STMN1 binding to the upstream region of the mouse *Cyp11a1* gene.** (a) Representation of potential STMN1-binding motifs on the mouse *Cyp11a1* promoter and five primer pairs designed for ChIP quantitative PCR; (b) STMN1 bound the *Cyp11a1* promoter. Normalized inputs of GCs chromatin DNA were pulled down by STMN1 or negative IgG antibodies. The DNA template was amplified by PCR using primer pairs 1-5 against the possible binding sites. (c) Ratios of the “ChIP band” to the “input band”. IgG controls were normalized to 1.0. ChIP experiments and PCR reactions were repeated twice, and quantified as the mean ± SEM.


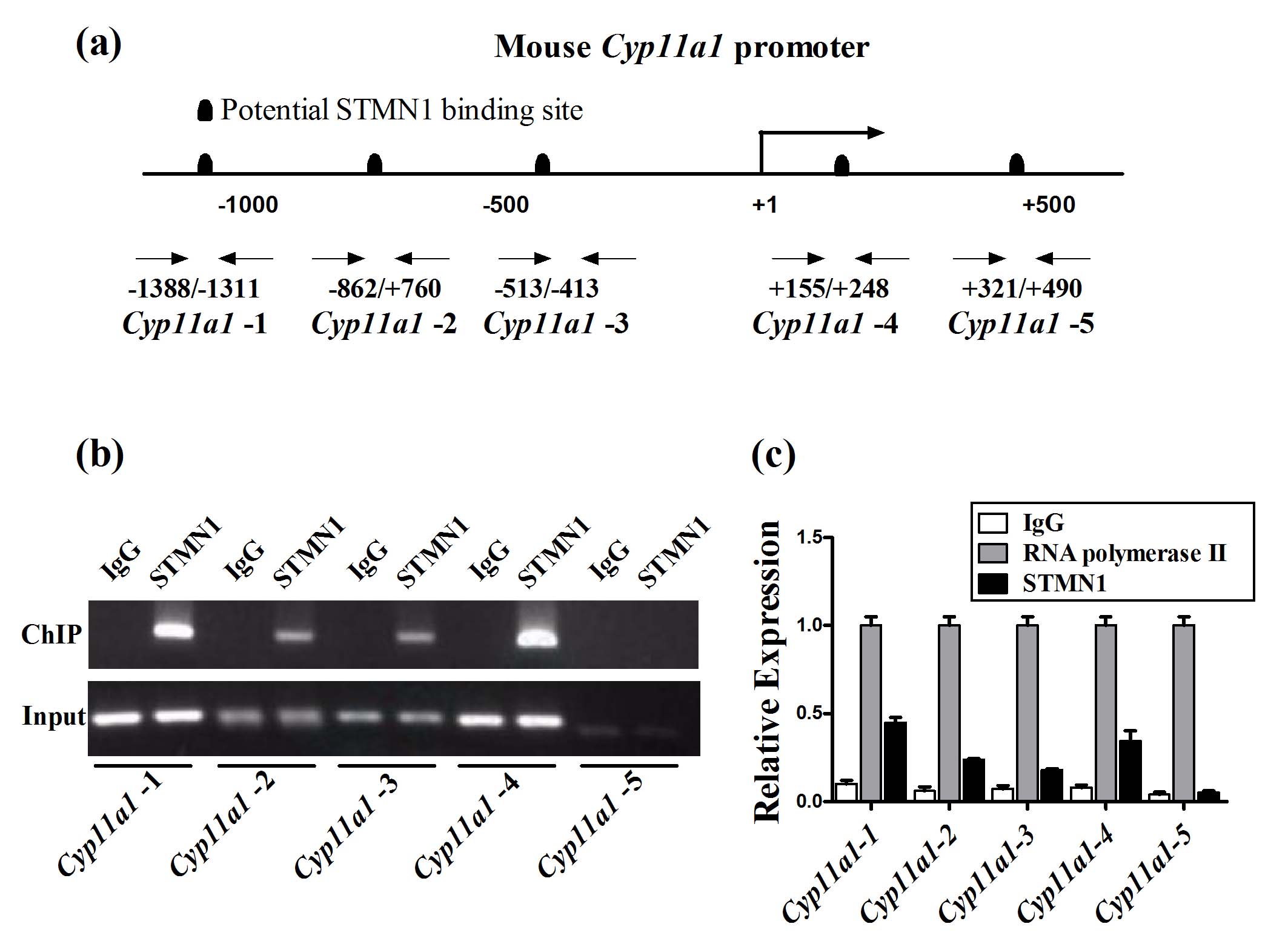

Supplement: Supplementary Information [file srep26691-s1.doc]
